# Non-coding RNAs enter mitosis: functions, conservation and implications

**DOI:** 10.1186/1747-1028-6-6

**Published:** 2011-02-28

**Authors:** Jun Wei Pek, Toshie Kai

**Affiliations:** 1 Department of Biological Sciences and Temasek Life Sciences Laboratory, 1 Research Link, The National University of Singapore, 117604, Singapore

## Abstract

Nuage (or commonly known as chromatoid body in mammals) is a conserved germline-specific organelle that has been linked to the Piwi-interacting RNA (piRNA) pathway. piRNAs are a class of gonadal-specific RNAs that are ~23-29 nucleotides in length and protect genome stability by repressing the expression of deleterious retrotransposons. More recent studies in *Drosophila *have implicated the piRNA pathway in other functions including canalization of embryonic development, regulation of maternal gene expression and telomere protection. We have recently shown that Vasa (known as Mouse Vasa Homolog in mouse), a nuage component, plays a mitotic role in promoting chromosome condensation and segregation by facilitating robust chromosomal localization of condensin I in the *Drosophila *germline. Vasa functions together with Aubergine (a PIWI family protein) and Spindle-E/mouse TDRD-9, two other nuage components that are involved in the piRNA pathway, therefore providing a link between the piRNA pathway and mitotic chromosome condensation. Here, we propose and discuss possible models for the role of Vasa and the piRNA pathway during mitosis. We also highlight relevant studies implicating mitotic roles for RNAs and/or nuage in other model systems and their implications for cancer development.

## Introduction

Germline granules were first described in rat spermatids more than 100 years ago and were subsequently named "chromatoid bodies" in mammalian cells [1,2]. They were later found to be widely-conserved in germline cells of many animals, where they are referred to as "nuage" and "P granules" in *Drosophila melanogaster *and *Caenorhabditis elegans*, respectively [3,4]. Under the electron microscope, germline granules appear as electron-dense fibrous structures, are not bound by any membrane and localize to the cytoplasmic peri-nuclear region [3]. Since their discovery, germline granules have remained mysterious due to the fact that their precise function has not been identified.

Recent studies in *Drosophila *have linked these germline granules (hereafter referred to as nuage) to a novel class of small non-coding RNAs known as Piwi-interacting RNAs (piRNAs). piRNAs are a class of gonadal-specific RNAs that are ~23-29 nucleotides in length and produced in a Dicer-independent manner [5-9]. They are mainly derived from transposons or repetitive sequences that are clustered in the peri-centromeric and sub-telomeric regions of the chromosome [10,11]. Interestingly, many proteins that are required for the biogenesis of piRNAs are found to localize to the nuage. For example, in *Drosophila*, the PIWI subfamily proteins, Aubergine and Argonaute3, which bind piRNAs, are components of the nuage [11-14]. In mouse, the PIWI family proteins, MILI and MIWI, also localize to the chromatoid body [5]. In the *Drosophila *nuage, Aubergine and Argonaute3 are believed to function in a secondary piRNA amplification pathway known as the "ping-pong" cycle in germline cells [11,15]. Other germline piRNA pathway proteins, such as Vasa (Mouse Vasa Homolog), Spindle-E (mouse TDRD-9), Krimper, Maelstrom (mouse Maelstrom), Cutoff (yeast Rai1) and Tejas (mouse TDRD-5 and TDRD-7), also localize to nuage although their exactly molecular function in piRNA processing remains unknown [16-20]. Of note, some piRNA pathway components (Piwi, Rhino and Armitage/mouse MOV10L1) do not localize to nuage, but play a role in the primary production of germline piRNAs [21-24], and Maelstrom has an additional role in the nucleus to regulate germline stem cell differentiation [25].

The first identified role for the piRNA pathway is to repress deleterious retrotransposons and the repeated *Stellate *elements in the *Drosophila *germline [8,26,27]. This process appears to occur in part at the transcriptional level involving Piwi as the effector protein to promote heterochromatin formation [28-30]. It has also been proposed that post-transcriptional silencing of retrotransposons occurs at "pi-bodies" - cytoplasmic nuage in conjunction with Processing body components [31]. As germline cells function to give rise to the next generation, it is not difficult to imagine that they would adopt multiple mechanisms to protect their genome integrity.

Recent studies have shown that the piRNA pathway functions not only to repress transposons but also to regulate embryonic development and telomere protection. Studies of Heat Shock Protein 90 (Hsp90) and Piwi have linked the piRNA pathway to the canalization of embryonic development by consecutively suppressing genetic variation via an epigenetic mechanism and silencing transposon activity [32,33]. The piRNA pathway has also been shown to regulate deadenylation and decay of maternal mRNAs in the embryo [34], therefore implicating additional functions of the piRNA pathway outside the germline. Besides regulating gene expression, another emerging role of the piRNA pathway is to protect the telomeres by regulating the telomere capping complex and telomere length [35,36]. Therefore, we are only beginning to understand the functional roles of the piRNA pathway in various biological processes.

## Discussion

### Nuage and the piRNA pathway in mitotic chromosome condensation and segregation

We recently reported that in *Drosophila*, Vasa, a piRNA pathway component, promotes mitotic chromosome condensation and segregation by facilitating robust chromosomal localization of two condensin I components: CAP-H (also known as Barren in *Drosophila*) and CAP-D2 [37]. Condensin complexes are major regulators of mitotic chromosomes that promote robust chromosome condensation and segregation [38,39]. *vasa *encodes a RNA helicase that is specifically expressed in germline cells [12,40]. Since its discovery in *Drosophila*, Vasa has been shown to be involved in various biological processes, including germline stem cell differentiation, piRNA-mediated transposon silencing, pole-plasm assembly, and germline proliferation [17,40,41]. In *Drosophila*, Vasa functions in part by interacting with eukaryotic initiation factor 5B (eIF5B) to promote the expression of proteins required for proper differentiation including Mei-P26 [41]. In mouse, the Mouse Vasa Homolog has also been shown to regulate germline proliferation and piRNA-mediated transposon silencing [2,5,12].

**Figure 1:**
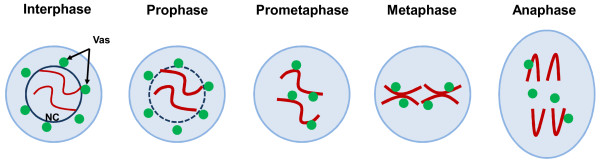
**The dynamic localization of Vasa during mitosis in *Drosophila *germline stem cells**. During interphase and prophase, Vasa (green) localizes as peri-nuclear bodies in the cytoplasm (nuage). When the nuclear envelope (dark blue line) begins to break down during prometaphase, Vasa gets access to the chromosomes (red) and localizes as peri-chromosomal bodies. This continues until metaphase. At anaphase, some Vasa bodies remain near segregation chromosomes, while the other bodies are displaced in between the segregating sister chromatids. NC: nucleus, Vas: Vasa.

We found that Vasa functions in an eIF5B-independent manner in promoting mitotic chromosome condensation and segregation in *Drosophila *[37]. During mitosis, Vasa forms mitotic bodies with Aubergine and Spindle-E (two other piRNA pathway proteins) and binds to piRNA-generating chromosomal loci at the peri-centromeric regions (Figure 1). In the piRNA pathway mutants, *aubergine *and *spindle-E*, chromosomal condensation and segregation were defective and concomitantly mitotic localization of Vasa is abolished. This suggests a link between the piRNA pathway and mitotic chromosome configuration. Furthermore, Vasa associates with CAP-H and CAP-D2, implying a direct role for Vasa in regulating their chromosomal localizations.

**Figure 2:**
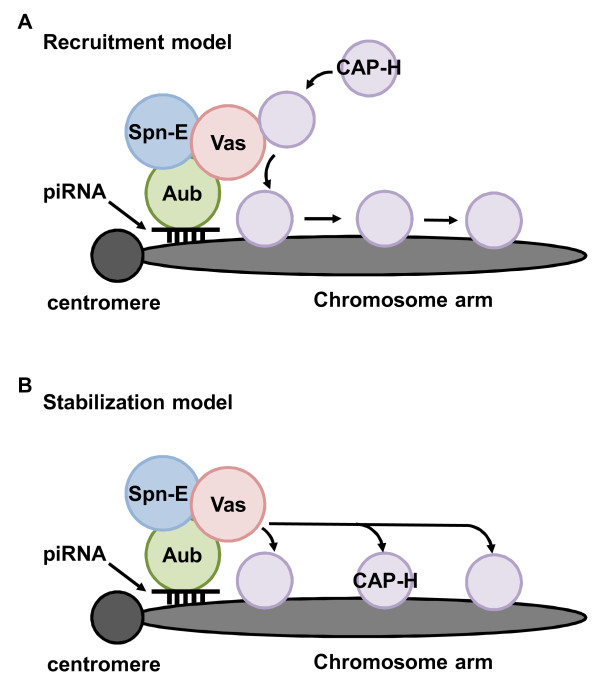
**Proposed mechanisms by which Vasa regulates condensin I (CAP-H) localization**. (A) The Recruitment model: Vasa functions primarily to recruit CAP-H at the peri-centromeric regions. After CAP-H loading, it spreads to the distal chromosome arms. (B) The Stabilization model: At the peri-centromeric region, Vasa functions in a yet unknown mechanism to promote a stable association of CAP-H to the mitotic chromosomes. Aub: Aubergine, Spn-E: Spindle-E,

How does Vasa regulate condensin I localization? We hypothesize that Vasa may directly function to promote recruitment of condensin I during mitosis (Figure 2). This model is favored because it has been shown that during mitosis, CAP-H loading begins at centromeric regions and subsequently spreads distally towards the chromosomal arms [42]. During mitosis, Vasa localizes near peri-centromeric piRNA-generating loci possibly via Aubergine-bound piRNAs. This localized region consisting of Vasa (and also Spindle-E) promotes recruitment of CAP-H to the chromosomes. Our studies on the genetic and physical interaction between Vasa and condensin I components also support this model. It would be interesting to test the functional significance of the Vasa-CAP-H interaction to further refine this model. Another model which may also seem possible but is less supported is the idea that the Vasa/Spindle-E/Aubergine-piRNA complex may localize to peri-centromeric piRNA-generating loci and stabilize CAP-H chromosomal localization (Figure 2). This model supposes a long-range action of Vasa on chromosomes by an unknown mechanism.

The involvement of Vasa and the piRNA pathway in germline mitotic chromosome regulation raises an intriguing question of whether an analogous pathway performs the same function in the soma. Although experimental evidence is lacking, recent studies suggest that an analogous somatic pathway may operate to promote condensin I localization. Somatic cells contain endogenous small interfering RNAs (endo-siRNAs) which function similarly to piRNAs in repressing retrotransposons [5]. Interestingly, some endo-siRNAs are derived from the same peri-centromeric loci that generate piRNAs [5], suggesting that these loci, together with the endo-siRNA pathway components, may collaborate in promoting condensin I recruitment in somatic cells. Another implication comes from a large-scale screen of localized RNAs in *Drosophila *embryos. A few retrotransposons (*copia, Doc*, and *Ste12DOR*) were identified to be localized onto mitotic chromosomes in developing embryos [43,44]. Therefore, it would be of interest to examine if the endo-siRNA pathway and a Vasa-related protein regulate condensin I in somatic cells.

### Mitotic roles of germline granules and/or RNAs in other organisms

Multiple lines of evidence for the role of germline granules and/or RNAs during mitosis have emerged over the past few years. Studies in *C. elegans *first demonstrated that some of the P granule (nuage equivalent) components, including Argonaute CSR-1, RNA-dependent RNA polymerase EGO-1, Dicer-related helicase DRH-3 and Tudor-domain protein EKL-1, promote chromosome segregation during mitosis by regulating proper chromosome organization in germline cells [45,46]. These proteins may form a mitotic complex that binds to CSR-1-interacting small RNAs (22G-RNAs) and localizes to mitotic holocentric chromosomes at the 22G-RNA target loci. Therefore, the 22G-RNAs appear to mediate recruitment of proteins to the mitotic chromosomes to promote chromosome segregation. These observations are intriguingly similar to what we see in *Drosophila*, where we observe that the RNA helicase Vasa, the Tudor-domain-containing RNA helicase Spindle-E and the PIWI subfamily protein Aubergine all localize to mitotic chromosomes [37]. Despite the differences between the two systems, it seems that, in general, small non-coding RNAs and germline granules appear to play important roles in organizing chromosomal configuration in germline cells during cell division.

In addition to the dynamic localization of nuage/P granules during mitosis, cell cycle-dependent expression of non-coding RNAs has also been reported in *Schizosaccharomyces pombe *(fission yeast) and mouse [47-49]. In mouse, it was reported that satellite repeats from the pericentric heterochromatin were transcribed during mitosis, suggesting a mitotic role for such non-coding RNAs [47]. Interestingly, non-coding centromeric satellite repeats are shown to be a component of the chromosome passenger complex (CPC) and potentiates Aurora A kinase activity in murine cultured cells [50]. In yeast, centromeric repeats are transcribed during the S phase before the loading of condensin, suggesting that such non-coding RNAs may promote recruitment of condensin during mitosis to silence the expression of centromeric repeats in a cell cycle-dependent manner [48].

RNAs also appear to have some function not only in chromosomal configuration, but also in spindle formation. Studies in *Xenopus laevis *and human cell lines have shown that mRNAs localize to mitotic spindles and appear to have a function independent of protein coding during mitosis: regulating spindle assembly [51,52]. This further implicates the functional roles of RNAs, either short non-coding RNAs or mRNAs, in various processes, including but not limited to chromosome organization and spindle assembly, during mitosis.

### Disease implications

Piwi, a founding member of the piRNA pathway, was originally found to regulate stem cell division in the *Drosophila *germline [53]. It was also reported that human and mouse Piwi homologues, Hiwi, PiwiL2, and PiwiL2-like proteins, are expressed in certain human and mouse stem cells and tumors [54-57], raising the possibility that the development of cancer may be linked to the piRNA pathway and stem cells. Using the *Drosophila *brain as a model system, a recent study indeed demonstrated that ectopic expression of germline and piRNA pathway genes are responsible for formation and development of brain tumors [58]. Although the mechanisms of how the piRNA pathway promotes tumorigenesis remain unknown, these data highlight the importance of understanding the role of the piRNA pathway during somatic cell division, with the idea that this pathway may be a target for cancer therapeutics.

## Conclusions

Our recent work on the role of Vasa and the piRNA pathway in promoting chromosome condensation and segregation during mitosis provides one example of a mitotic role for non-coding RNAs. Similar roles for such non-coding RNAs have also been described in various model organisms, including yeast, *C. elegans*, *Xenopus *and mouse. Future studies would likely uncover more examples of the fascinating interplay between RNAs and the cell cycle.

## List of abbreviations used

mRNA: messenger RNA; piwi: P-element induced wimpy testis.

## Competing interests

The authors declare that they have no competing interests.

## Authors' contributions

JWP and TK drafted, read and approved the manuscript.

## References

[B1] Yokota S Historical survey on chromatoid body research Acta Histochem Cytochem 2008 41 65 82 10.1267/ahc.08010 18787638 PMC2532602

[B2] Kotaja N Sassone-Corsi P The chromatoid body: a germ-cell-specific RNA-processing centre Nat Rev Mol Cell Biol 2007 8 85 90 10.1038/nrm2081 17183363

[B3] Eddy EM Germ plasm and the differentiation of the germ line Int Rev Cytol 1975 43 229 281 10.1016/S0074-7696(08)60070-4 770367

[B4] Updike D Strome S P granule assembly and function in *Caenorhabditis elegans *germ cells J Androl 2010 31 53 60 10.2164/jandrol.109.008292 19875490 PMC2905540

[B5] Saito K Siomi MC Small RNA-mediated quiescence of transposable elements in animals Dev Cell 2010 19 687 697 10.1016/j.devcel.2010.10.011 21074719

[B6] Senti KA Brennecke J The piRNA pathway: a fly's perspective on the guardian of the genome Trends Genet 2010 26 499 509 10.1016/j.tig.2010.08.007 20934772 PMC4988489

[B7] Khurana JS Theukauf W piRNAs, transposon silencing, and Drosophila germline development J Cell Biol 2010 191 905 913 10.1083/jcb.201006034 21115802 PMC2995163

[B8] Vagin VV Sigova A Li C Seitz H Gvozdev V Zamore PD A distinct small RNA pathway silences selfish genetic elements in the germline Science 2006 313 320 324 10.1126/science.1129333 16809489

[B9] Aravin AA Lagos-Quintana M Yalcin A Zavolan M Marks D Snyder B Gaasterland T Meyer J Tuschl T The small RNA profile during *Drosophila melanogaster *development Dev Cell 2003 5 337 350 10.1016/S1534-5807(03)00228-4 12919683

[B10] Saito K Nishida KM Mori T Kawamura Y Miyoshi K Nagami T Siomi T Siomi MC Specific association of Piwi with rasiRNAs derived from retrotransposon and heterochromatic regions in the *Drosophila *genome Genes Dev 2006 20 2214 2222 10.1101/gad.1454806 16882972 PMC1553205

[B11] Brennecke J Aravin AA Stark A Dus M Kellis M Sachidanandam R Hannon GJ Discrete small RNA-generating loci as master regulators of transposon activity in *Drosophila* Cell 2007 128 1089 1103 10.1016/j.cell.2007.01.043 17346786

[B12] Arkov AL Ramos A Building RNA-granules: insight from the germline Trends Cell Biol 2010 20 482 490 10.1016/j.tcb.2010.05.004 20541937 PMC2929181

[B13] Nishida KM Saito K Mori T Kawamura Y Nagami-Okada T Inagaki S Siomi H Siomi MC Gene silencing mechanisms mediated by Aubergine piRNA complexes in *Drosophila *male gonad RNA 2007 13 1911 1922 10.1261/rna.744307 17872506 PMC2040086

[B14] Nagao A Mituyama T Huang H Chen D Siomi MC Siomi H Biogenesis pathways of piRNAs loaded onto AGO3 in the *Drosophila *testis RNA 2010 16 2503 2515 10.1261/rna.2270710 20980675 PMC2995411

[B15] Gunawardane LS Saito K Nishida KM Miyoshi K Kawamura Y Nagami T Siomi H Siomi MC A slicer-mediated mechanism for repeat-associated siRNA 5' end formation in *Drosophila* Science 2007 315 1587 1590 10.1126/science.1140494 17322028

[B16] Vagin VV Klenov MS Kalmykova AI Stolyarenko AD Kotelnikov RN Gvozdev VA The RNA interference proteins and *vasa *locus are involved in the silencing of retrotransposons in the female germline of *Drosophila melanogaster* RNA Biol 2004 1 54 58 17194939

[B17] Lim AK Kai T Unique germ-line organelle, nuage, functions to repress selfish genetic elements in *Drosophila melanogaster* Proc Natl Acad Sci USA 2007 104 6714 6719 10.1073/pnas.0701920104 17428915 PMC1871851

[B18] Patil VS Kai T Repression of retroelements in *Drosophila *germline via piRNA pathway by the Tudor domain protein Tejas Curr Biol 2010 20 724 730 10.1016/j.cub.2010.02.046 20362446

[B19] Findley SD Tamanaha M Clegg NJ Ruohola-Baker H Maelstrom, a *Drosophila *spindle-class gene, encodes a protein that colocalizes with Vasa and RDE1/AGO1 homolog, Aubergine, in nuage Development 2003 130 859 871 10.1242/dev.00310 12538514

[B20] Chen Y Pane A Schupbach T *cutoff *and *aubergine *mutations result in retrotransposon upregulation and checkpoint activation in *Drosophila* Curr Biol 2007 17 637 642 10.1016/j.cub.2007.02.027 17363252 PMC1905832

[B21] Saito K Inagaki S Mituyama T Kawamura Y Ono Y Sakota E Kotani H Asai K Siomi H Siomi MC A regulatory circuit for *piwi *by the large Maf gene *traffic jam *in *Drosophila* Nature 2009 461 1296 1299 10.1038/nature08501 19812547

[B22] Klattenhoff C Xi H Li C Lee S Xu J Khurana JS Zhang F Schultz N Koppetsch BS Nowosielska A Seitz H Zamore PD Weng Z Theurkauf WE The *Drosophila *HP1 homolog Rhino is required for transposon silencing and piRNA production by dual-strand clusters Cell 2009 138 1137 1149 10.1016/j.cell.2009.07.014 19732946 PMC2770713

[B23] Malone CD Brennecke J Dus M Stark A McCombie WR Sachidanandam R Hannon GJ Specialized piRNA pathways act in germline and somatic tissues of the *Drosophila *ovary Cell 2009 137 522 535 10.1016/j.cell.2009.03.040 19395010 PMC2882632

[B24] Li C Vagin VV Lee S Xu J Ma S Xi H Seitz H Horwich MD Syrzycka M Honda BM Kittler EL Zapp ML Klattenhoff C Schulz N Theurkauf WE Weng Z Zamore PD Collapse of germline piRNAs in the absence of Argonaute3 reveals somatic piRNAs in flies Cell 2009 137 509 521 10.1016/j.cell.2009.04.027 19395009 PMC2768572

[B25] Pek JW Lim AK Kai T *Drosophila *Maelstrom ensures proper germline stem cell lineage differentiation by repressing *microRNA-7* Dev Cell 2009 17 417 424 10.1016/j.devcel.2009.07.017 19758565

[B26] Aravin AA Klenov MS Vagin VV Bantignies F Cavalli G Gvozdev VA Dissection of a natural RNA silencing process in the *Drosophila melanogaster *germ line Mol Cell Biol 2004 24 6742 6750 10.1128/MCB.24.15.6742-6750.2004 15254241 PMC444866

[B27] Aravin AA Naumova NM Tulin AV Vagin VV Rozovsky YM Gvozdev VA Double-stranded RNA-mediated silencing of genomic tandem repeats and transposable elements in the *D. melanogaster *germline Curr Biol 2001 11 1017 1027 10.1016/S0960-9822(01)00299-8 11470406

[B28] Yin H Lin H An epigenetic activation role of Piwi and a Piwi-associated piRNA in *Drosophila melanogaster* Nature 2007 450 304 308 10.1038/nature06263 17952056

[B29] Brower-Toland B Findley SD Jiang L Liu L Yin H Dus M Zhou P Elgin SC Lin H *Drosophila *PIWI associates with chromatin and interacts with HP1a Genes Dev 2007 21 2300 2311 10.1101/gad.1564307 17875665 PMC1973144

[B30] Klenov MS Lavrov SA Stolyarenko AD Ryazansky SS Aravin AA Tuschl T Gvozdev VA Repeat-associated siRNAs cause chromatin silencing of retransposons in the *Drosophila melanogaster *germline Nucleic Acids Res 2007 35 5430 5438 10.1093/nar/gkm576 17702759 PMC2018648

[B31] Lim AK Tao L Kai T piRNAs mediate posttranscriptional retroelement silencing and localization to pi-bodies in the *Drosophila *germline J Cell Biol 2009 186 333 342 10.1083/jcb.200904063 19651888 PMC2728408

[B32] Specchia V Piacentini L Tritto P Fanti L D'Alessandro R Palumbo G Pimpinelli S Bozzetti MP Hsp90 prevents phenotypic variation by suppressing the mutagenic activity of transposons Nature 2010 463 662 665 10.1038/nature08739 20062045

[B33] Gangaraju VK Yin H Weiner MM Wang J Huang XA Lin H *Drosophila *Piwi functions in Hsp90-mediated suppression of phenotypic variation Nat Genet 2011 43 153 158 10.1038/ng.743 21186352 PMC3443399

[B34] Rouget C Papin C Boureux A Meunier AC Franco B Robine N Lai EC Pelisson A Simonelig M Maternal mRNA deadenylation and decay by the piRNA pathway in the early *Drosophila *embryo Nature 2010 467 1128 1132 10.1038/nature09465 20953170 PMC4505748

[B35] Khurana JS Xu J Weng Z Theurkauf WE Distinct functions for the *Drosophila *piRNA pathway in genome maintenance and telomere protection PLoS Genet 2010 6 e1001246 10.1371/journal.pgen.1001246 21179579 PMC3003142

[B36] Savitsky M Kwon D Georgiev P Kalmykova A Gvozdev V Telomere elongation is under the control of the RNAi-based mechanism in the *Drosophila *germline Genes Dev 2006 20 345 354 10.1101/gad.370206 16452506 PMC1361705

[B37] Pek JW Kai T A role for Vasa in regulating mitotic chromosome condensation in *Drosophila* Curr Biol 2011 21 39 44 10.1016/j.cub.2010.11.051 21185189

[B38] Hirano T Condensins: organizing and segregating the genome Curr Biol 2005 15 R265 275 10.1016/j.cub.2005.03.037 15823530

[B39] Hudson DF Marshall KM Earnshaw WC Condensin: architect of mitotic chromosomes Chromosome Res 2009 17 131 144 10.1007/s10577-008-9009-7 19308696

[B40] Styhler S Nakamura A Swan A Suter B Lasko P *vasa *is required for GURKEN accumulation in the oocyte, and is involved in oocyte differentiation and germline cyst development Development 1998 125 1569 1578 9521895 10.1242/dev.125.9.1569

[B41] Liu N Han H Lasko P Vasa promotes *Drosophila *germline stem cell differentiation by activating mei-P26 translation by directly interacting with a (U)-rich motif in its 3'UTR Genes Dev 2009 23 2742 2752 10.1101/gad.1820709 19952109 PMC2788330

[B42] Oliveira RA Heidmann S Sunkel CE Condensin I binds chromatin early in prophase and displays a highly dynamic association with *Drosophila *mitotic chromosomes Chromosoma 2007 116 259 274 10.1007/s00412-007-0097-5 17318635

[B43] Lécuyer E Yoshida H Parthasarathy N Alm C Babak T Cerovina T Hughes TR Tomancak P Krause HM Global analysis of mRNA localization reveals a prominent role in organizing cellular architecture and function Cell 2007 131 174 187 17923096 10.1016/j.cell.2007.08.003

[B44] Lécuyer E Yoshida H Krause HM Global implications of mRNA localization pathways in cellular organization Curr Opin Cell Biol 2009 21 409 415 19249199 10.1016/j.ceb.2009.01.027

[B45] van Wolfswinkel JC Claycomb JM Batista PJ Mello CC Berezikov E Ketting RF CDE-1 affects chromosome segregation through uridylation of CSR-1-bound siRNAs Cell 2009 139 135 148 10.1016/j.cell.2009.09.012 19804759

[B46] Claycomb JM Batista PJ Pang KM Gu W Vasale JJ van Wolfswinkel JC Chaves DA Shirayama M Mitani S Ketting RF Conte D Jr Mello CC The argonaute CSR-1 and its 22G-RNA cofactors are required for holocentric chromosome segregation Cell 2009 139 123 134 10.1016/j.cell.2009.09.014 19804758 PMC2766185

[B47] Lu J Gilbert DM Proliferation-dependent and cell cycle-regulated transcription of mouse pericentric heterochromatin J Cell Biol 2007 179 411 421 10.1083/jcb.200706176 17984319 PMC2064789

[B48] Chen ES Zhang K Nicolas E Cam HP Zofall M Grewal SIS Cell cycle control of centromeric repeat transcription and heterochromatin assembly Nature 2008 451 734 737 10.1038/nature06561 18216783

[B49] Lu J Gilbert DM Cell cycle regulated transcription of heterochromatin in mammals vs. fission yeast Cell Cycle 2008 7 1907 1910 10.4161/cc.7.13.6206 18604169 PMC2710769

[B50] Ferri F Bouzinba-Segard H Velasco G Hube F Francastel C Non-coding murine centromeric transcripts associate with and potentiate Aurora B kinase Nucleic Acids Res 2009 37 5071 5080 10.1093/nar/gkp529 19542185 PMC2731909

[B51] Blower MD Nachury M Heald R Weis K A Rae1-containing ribonucleoprotein complex is required for mitotic spindle assembly Cell 2005 121 223 234 10.1016/j.cell.2005.02.016 15851029

[B52] Blower MD Feric E Weis K Hearld R Genome-wide analysis demonstrates conserved localization of messenger RNAs to mitotic microtubules J Cell Biol 2007 179 1365 1373 10.1083/jcb.200705163 18166649 PMC2373496

[B53] Cox DN Chao A Lin H *piwi *encodes a nucleoplasmic factor whose activity modulates the number and division rate of germline stem cells Development 2000 127 503 514 10631171 10.1242/dev.127.3.503

[B54] Sharma AK Nelson MC Brandt JE Wessman M Mahmud N Weller KP Hoffman R Human CD34+ stem cells express the *hiwi *gene, a human homologue of the *Drosophila *gene *piwi* Blood 2001 97 426 434 10.1182/blood.V97.2.426 11154219

[B55] Ye Y Yin D-T Chen L Zhou Q Shen R He G Yan Q Tong Z Issekutz AC Shapiro CL Barsky SH Lin H Li J-J Gao J-X Identification of Piwil2-Like (PL2L) proteins that promote tumorigenesis PLoS ONE 2010 5 e13406 10.1371/journal.pone.0013406 20975993 PMC2958115

[B56] Qiao D Zeeman A-M Deng W Looijenga LHJ Lin H Molecular characterization of *hiwi*, a human member of the *piwi *gene family whose overexpression is correlated to seminomas Oncogene 2002 21 3988 3999 10.1038/sj.onc.1205505 12037681

[B57] Wu Q Ma Q Shehadeh LA Wilson A Xia L Yu H Webster KA Expression of the Argonaute protein PiwiL2 in adult mouse mesenchymal stem cells Biochem Biophys Res Commun 2010 396 915 920 10.1016/j.bbrc.2010.05.022 20460113 PMC3151571

[B58] Janic A Mendizabal L Llamazares S Rossell D Gonzalez C Ectopic expression of germline genes drives malignant brain tumor growth in *Drosophila* Science 2010 330 1824 1827 10.1126/science.1195481 21205669

